# Supplementary material for: A conserved interaction of the dynein light intermediate chain with dynein-dynactin effectors necessary for processivity
Source: Nat Commun. 2018 Mar 7;9:986. doi: 10.1038/s41467-018-03412-8 (PMC5841405; doi:10.1038/s41467-018-03412-8)
Supplement: Supplementary file 1 — Supplementary Information [file 41467_2018_3412_MOESM1_ESM.pdf]

A Conserved Interaction of the Dynein Light Intermediate Chain with Dynein-Dynactin Effectors Necessary for Processivity

Lee et al.

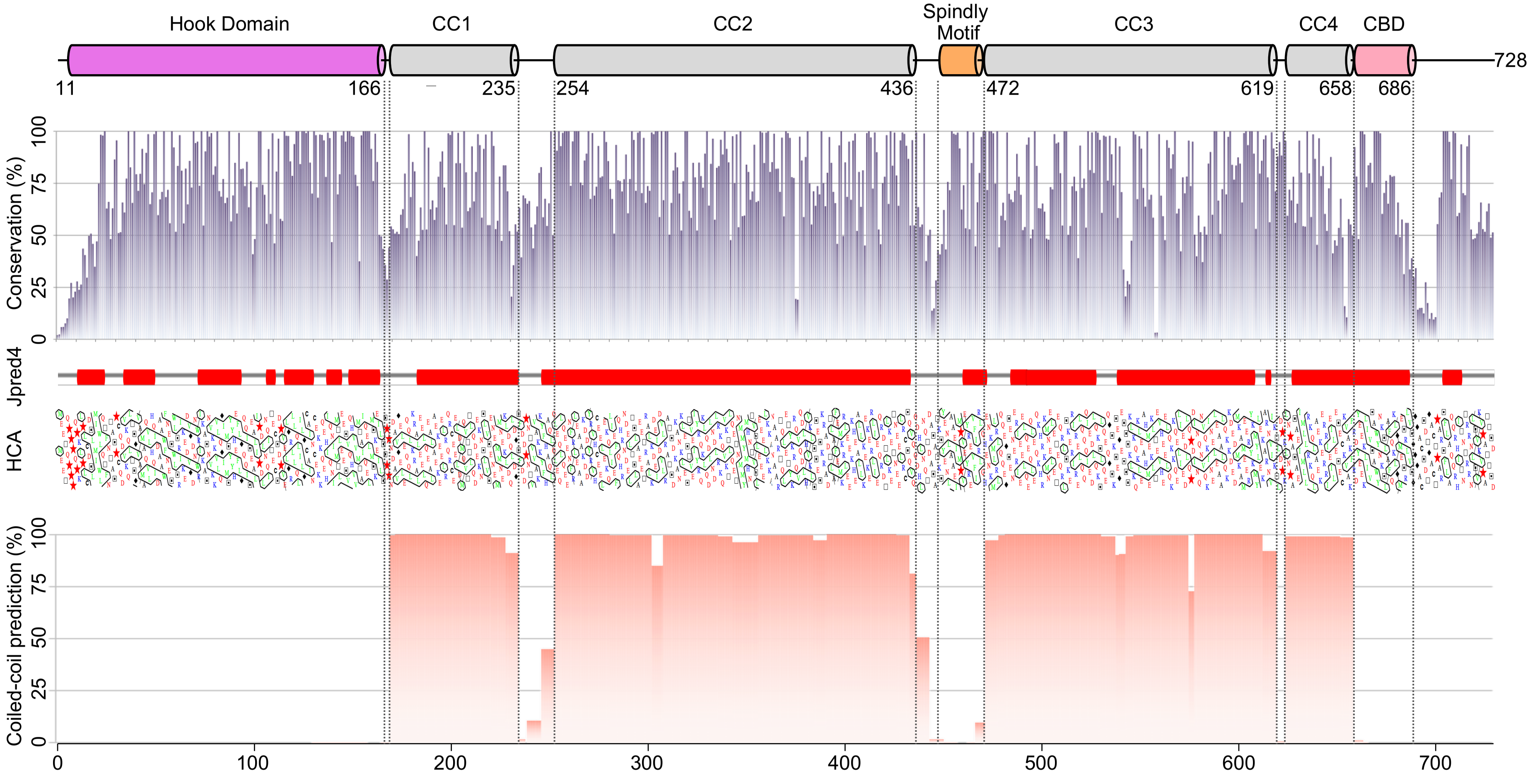

Hook Domain
CC1
CC2
Spindly Motif
CC3
CC4
CBD
11
166
235
254
436
472
619
658
686
728
Conservation (%)
Jpred4
HCA
Coiled-coil prediction (%)
0
25
50
75
100
200
300
400
500
600
700

Supplementary Figure 1 Domain architecture of Hook1. The domain architecture of human Hook1 was analyzed using several bioinformatics approaches, including: sequence conservation analysis with the program Scorecons[1], secondary structure prediction with the program Jpred4[2], hydrophobic cluster analysis with the program HCA[3], and coiled coil prediction with the program Coils[4]. Sequence conservation scores were calculated from an alignment of 96 Hook sequences from different species and isoforms. The resulting per-residue scores were then plotted against the human Hook1 sequence, i.e. residue insertions in other sequences (compared to human Hook1) are not shown. The secondary structure and coiled coil predictions suggest that Hook consists solely of α-helices, since not a single β-strand was predicted.

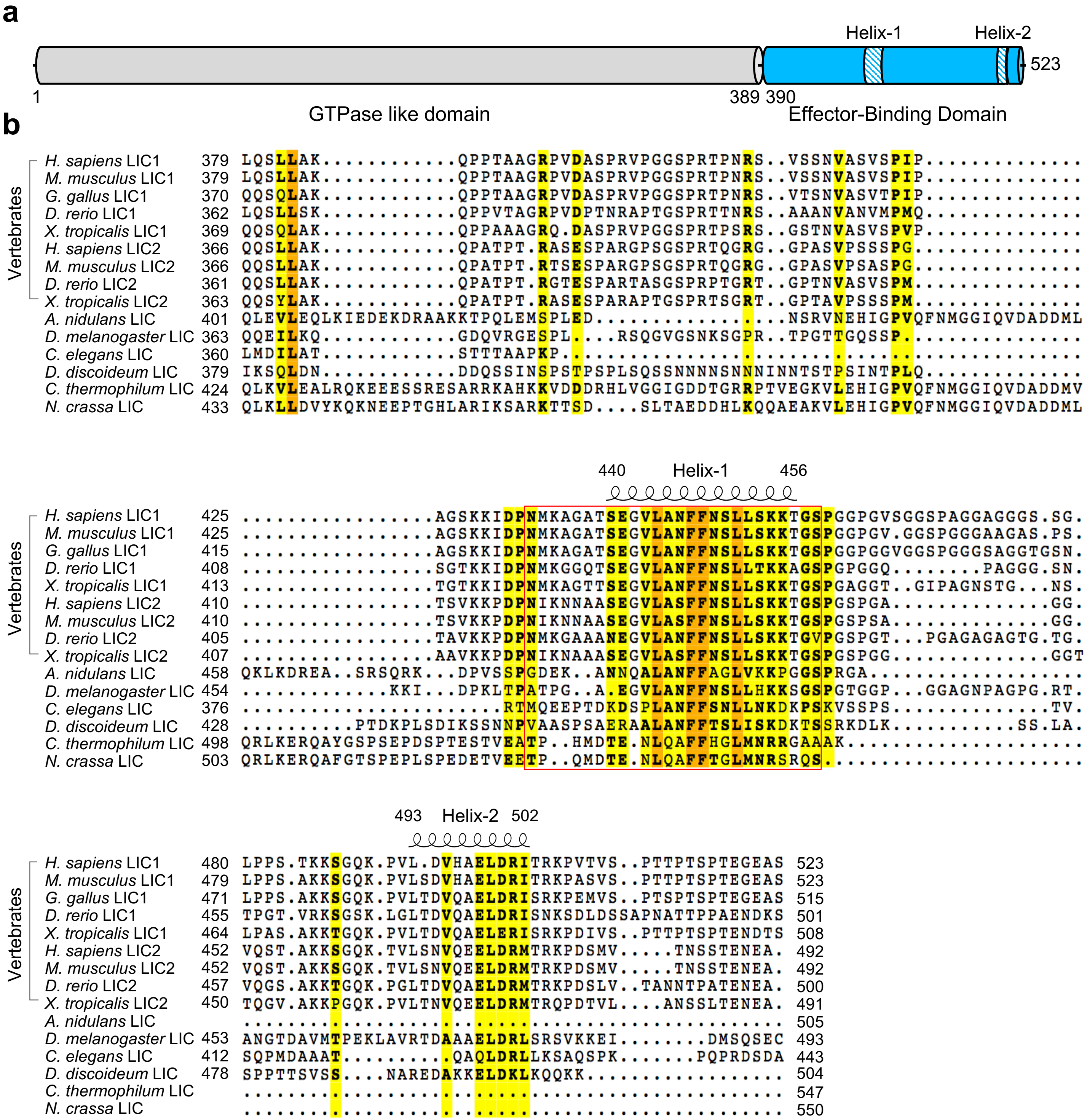

Supplementary Figure 2 Alignment of the Effector-Binding Domain of LIC sequences. (**a**) Domain diagram of human LIC1, showing the location of the predicted Helix-1 and Helix-2 within the C-terminal Effector-Binding Domain (EBD). (**b**) Sequence alignment of the EBD of LIC sequences from different species and isoforms. The name of each sequence includes the organism of origin. Yellow and orange backgrounds indicate 70% and 100% sequence conservation, respectively. The predicted Helix-1 and Helix-2 (depicted above the sequence alignment) coincide with regions of higher sequence conservation. The region corresponding to the Helix 1 ($LIC1_{433\text{-}458}$) peptide is contoured red.

Supplementary Table 1 Primers used in this study

Construct	Forward primer	Reverse primer
Hook1$_{11\text{-}166}$	5' cgcggatccgaaaatctgtatttccaatc cggcagcaagctgcccctgtgcgac	5' ctcgtcgacttagctcaatatttctttactcatcaactctt
Hook1$_{11\text{-}238}$	5' cgcggatccgaaaatctgtatttccaatc cggcagcaagctgcccctgtgcgac	5' ctcgtcgacttaatcatcaaaagagccatccaac
Hook1$_{1\text{-}239}$ GCN4	5' attgcggccgcggaaaatctgtatttccaa tccatggaggagacgcagccg<br>5' attcaattggaagacaaggttgaagaattg ctttc	5' tctcaattgtttcattggatcatcaaaaga gccatcca<br>5' ctcgtcgacttagagcttctttaatctggcaacctc attttcca
Hook1$_{11\text{-}443}$	5' cgcggatccgaaaatctgtatttccaatcc ggcagcaagctgcccctgtgcgac	5' ctcgtcgacttatgtttggtttaggtggtcct
Hook1$_{FL}$	5' acgcgtcgactggaaaatctgtatttccaa tccatggaggaaacccagcct	5' gctctagattatttttcgaactgcgggtggctccagcc accgtcagaagtggtagcaggg
Hook3$_{1\text{-}160}$	5' attgcggccgcggaaaatctgtatttc caat ccatgttcagcgtagagtcgctg	5' ctcgtcgactttttactcatcagctcttgaatgg
Hook3$_{1\text{-}143}$	5' attgcggccgcggaaaatctgtattt ccaatccatgttcagcgtagagtcgctg	5' ctcgtcgacttactccatcatcataatggcttg
MBP-LIC1$_{FL}$	5' cgcggatccggaaaatctgtatttccaatc catggcggccgtgggg	5' acggtcgactcaagaagcttctccttccgtaggagat
MBP-LIC1$_{1\text{-}461}$	5' cgcggatccggaaaatctgtatttccaatc catggcggccgtgggg	5' ctcgtcgacgcctcctggagagccagtcttttta
MBP-LIC1$_{1\text{-}437}$	5' cgcggatccggaaaatctgtatttccaatc catggcggccgtgggg	5' ctcgtcgactccagctttcatgtttggatcaattt
MBP-LIC1$_{FL}$ (F447A, F448A)	5' cagtctttttactcaacaaactgttggcggc atttgccagaacgccttcacttgtagc	5' gctacaagtgaaggcgttctggcaaatgccgccaa cagtttgttgagtaaaaagactg
LIC1$_{433\text{-}458}$	5' ggtggttgctcttccaacaacatgaaagct ggagctacaag	5' ctcgtagacttaagagccagtctttttactcaacaaa
Hook3$_{1\text{-}160}$ (A138D)	5' attcctccatcatcataatgtcttggatgtact cttgcttc	5' gaagcaagagtacatccaagacattatgatgatgga ggaat
Hook3$_{1\text{-}160}$ (M140D)	5' atgttgaacagattcctccatcatatcaatg gcttggatgtactcttgcttc	5' gaagcaagagtacatccaagccattgatatgatgg aggaatctgttcaacat
BICD2$_{1\text{-}98}$	5' cgcggatccgaaaacctgtattttcaggga atgtccgcgccgtcgga	5' ctcgtcgacttagctctcaccatcagcagcca
Spindly$_{1\text{-}142}$	5' cgcggatccgagaacctgtattttcaaagc atggaagcggacatcattacc	5' ctcgtcgacttagctcagcagttctttttgatgg

Supplementary References

1. Valdar, W.S. Scoring residue conservation. *Proteins* **48**, 227–241 (2002).
2. Lupas, A., Van Dyke, M., and Stock, J. Predicting coiled coils from protein sequences. *Science* **252**, 1162–1164 (1991).
3. Cole, C., Barber, J.D., and Barton, G.J. JPred4: a protein secondary structure prediction server. *Nucleic Acids Res.* **43**, W389–W394 (2015).
4. Gaboriaud, C., Bissery, V., Benchetrit, T., and Mornon, J.P. Hydrophobic cluster analysis: an efficient new way to compare and analyse amino acid sequences. *FEBS Lett.* **224**, 149–155 (1987).
